# P-78. Observational Comparison of In-Person vs. Remote Care for Bone and Joint Infections

**DOI:** 10.1093/ofid/ofae631.285

**Published:** 2025-01-29

**Authors:** Nupur Gupta, Seema Mehta Steinke, Sowmya Nanjappa, Nicholas D’Angelo, Devan Kowdley, Victoria R Wong, Rima Abdel-Massih, John W Mellors, Neel Shah

**Affiliations:** UPMC, Pittsburgh, Pennsylvania; University Of Pittsburgh Medical Center, Pittsbugh, Pennsylvania; UPMC, Pittsburgh, Pennsylvania; University of Pittsburgh School of Medicine, Pittsburgh, Pennsylvania; University of Pittsburgh School of Medicine, Pittsburgh, Pennsylvania; University of Pittsburgh School of Medicine, Pittsburgh, Pennsylvania; University of Pittsburgh Medical Center, Pittsburgh, PA; University of Pittsburgh School of Medicine, Pittsburgh, Pennsylvania; University of Pittsburgh Medical Center, Pittsburgh, PA

## Abstract

**Background:**

Telemedicine can overcome barriers to accessing subspecialty expertise including for infectious disease (ID). A common need for ID expertise is bone and joint infections (BJI), which are associated with substantial morbidity and mortality. Access to BJI expertise in underserved locations could be increased through ID telemedicine (Tele-ID). Here, we compare patients, and outcomes of Tele-ID vs in-person ID care of BJI.

**Table 1:**
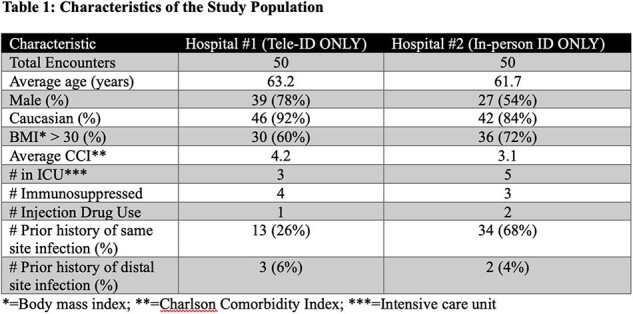
Characteristics of the Study Population

**Methods:**

We conducted a retrospective study of BJI patients who received Tele-ID (live audio-video) care at hospital #1 vs. in-person ID care at hospital #2 between 2015-2019. Both hospitals are affiliated with a larger academic institution and have care provided by the same ID physicians. Demographic data, comorbidities, diagnostic and microbiological data, treatment, and outcomes were assessed.

**Table 2:**
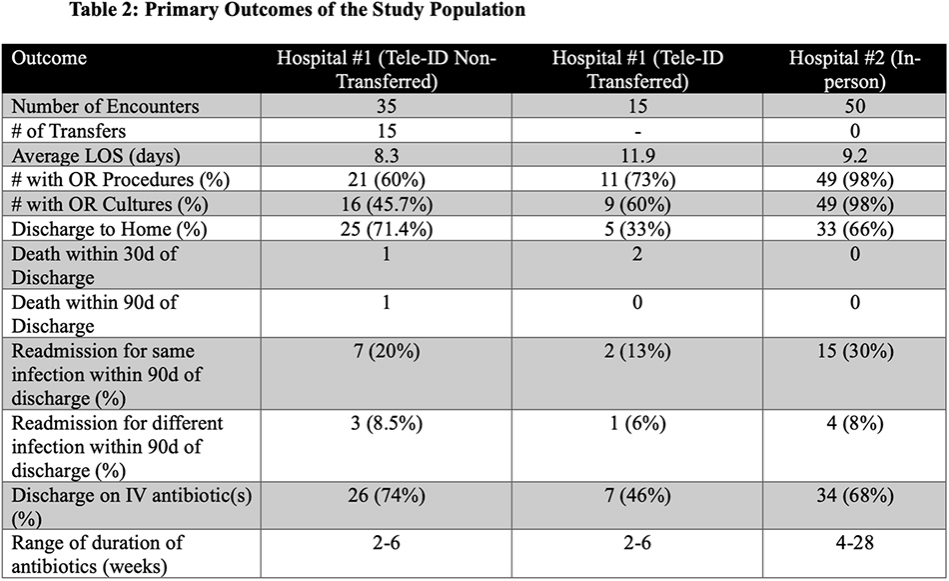
Primary Outcomes of the Study Population

**Results:**

Of the first 100 patients, half received Tele-ID care at hospital #1 and the other half received in-person ID care at hospital #2. Both groups had similar patient characteristics (Table 1), except for higher frequency of same site infection at hospital #2 (68 vs 26%). Patients cared for via Tele-ID were more often transferred to an alternative tertiary-care facility (30% vs. 0%) for surgical specialty services not available at hospital #1 (Table 2). For Tele-ID patients transferred vs in-person care, LOS was longer and discharge to home occurred less often compared with those not transferred. Readmission for the same infection within 30 days and mortality were not different (Table 2). The most common BJI managed by Tele-ID was diabetic foot osteomyelitis without any hardware, whereas joint infections with hardware were most commonly seen by in-person ID (Figure 1). Amongst patients with OR procedures, most OR cultures were negative, with *Staphylococcus aureus* being isolated most frequently at both hospitals (Figure 2).

**Figure 1:**
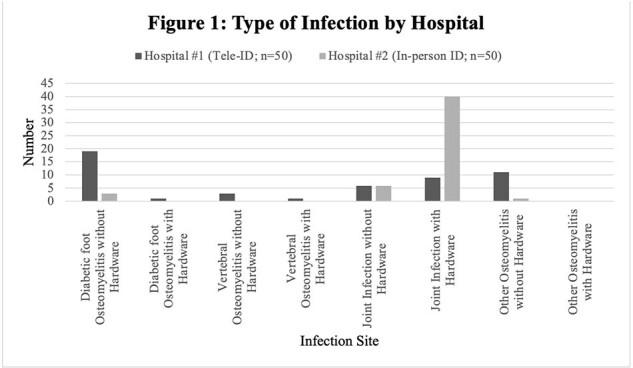
Type of Infection by Hospital

**Conclusion:**

This initial observational study suggests that clinical outcomes are similar for patients with BJI cared for by in-person vs. Tele-ID, although hospital transfer for surgical interventions was more common at the Tele-ID site. Further comparison in a larger number of patients at each hospital is ongoing. This preliminary experience suggests that Tele-ID could provide appropriate care for BJI where in-person ID expertise is not available.

**Figure 2:**
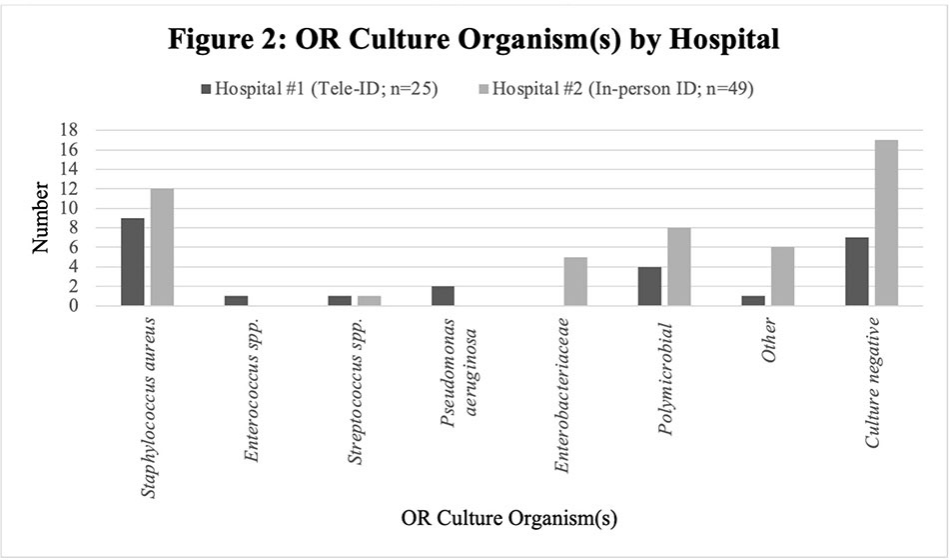
OR Culture Organism(s) by Hospital

**Disclosures:**

**Seema Mehta Steinke, MD, MS**, Pearl Diagnostics: Advisor/Consultant **Rima Abdel-Massih, MD**, Infectious Disease Connect: CEO & co-founder|Infectious Disease Connect: Ownership Interest **John W. Mellors, MD**, Infectious Disease (ID) Connect, Inc.: Share Options

